# Abducens Palsy Due to Cerebral Venous Sinus Thrombosis in a Patient with Heart Failure

**DOI:** 10.4274/tjo.94468

**Published:** 2015-08-05

**Authors:** Cem Özgönül, Osman Melih Ceylan, Fatih Mehmet Mutlu, Halil İbrahim Altınsoy, Mustafa Aparcı

**Affiliations:** 1 Anıttepe Military Dispensary, Clinic of Ophthalmology, Ankara, Turkey; 2 Ardahan Military Hospital, Clinic of Ophthalmology, Ardahan, Turkey; 3 Gülhane Military Medical Academy, Department of Ophthalmology, Ankara, Turkey; 4 Dünyagöz Hospital, Ankara, Turkey; 5 Etimesgut Military Hospital, Clinic of Cardiology, Ankara, Turkey

**Keywords:** Abducens palsy, cerebral venous sinus thrombosis, heart failure

## Abstract

Cerebral venous sinus thrombosis has a wide spectrum of presentation. The clinical manifestation depends on the location of the thrombus, its rate of progression, and the extent of venous collateralization. In this case report, we present the findings of cerebral venous sinus thrombosis presenting with abducens palsy and papilloedema in a patient with heart failure, an unusual etiology for cerebral venous sinus thrombosis.

## INTRODUCTION

Cerebral venous sinus thrombosis (CVST) was first described by Ribes in 1825 from the autopsy of a 45-year-old patient with headaches, epileptic seizures and delirium.^1^ Although rarely identified in the past, it is now easily diagnosed using currently available non-invasive, high-resolution imaging techniques.^2^ Clinical outcomes vary extremely, ranging from complete recovery to permanent and devastating neurological deficits.^3^ Magnetic resonance (MR) venography is the method of choice for the diagnosis of CVST.^4^ Up to 80% of CVST patients have the clinical features associated with increased thrombosis such as hypercoagulability, hormone replacement or oral contraceptive use, head trauma, dehydration, infection, malignancy, pregnancy, and increased blood viscosity.^2,5^

To the best of our knowledge, there are no previous reports of a case of abducens palsy due to CVST possibly resulting from heart failure with reduced left ventricular ejection fraction. In this report, we aim to emphasize the significance of CVST in the differential diagnosis and etiology of abducens palsy and the advantage of MR venography in the diagnosis.

## CASE REPORT

**Figure 1:**
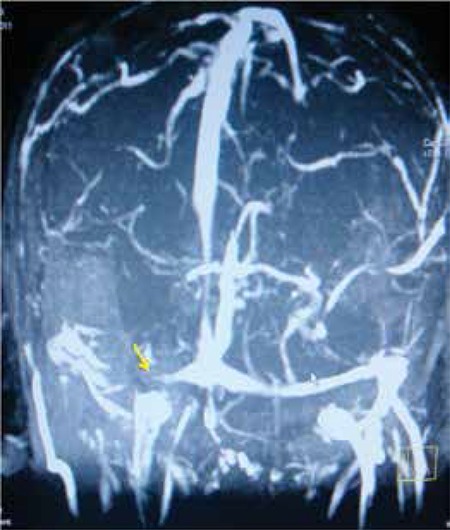
Loss of current signal consistent with thrombosis in the right transverse sinus, sigmoid sinus and proximal jugular vein (yellow arrow)

A 32-year-old patient presented with complaints of sudden-onset headache, nausea and vomiting, double vision and seizure. Clinical signs of abducens palsy were observed on neurological examination. The patient’s medical history included heart failure with sinus rhythm and reduced left ventricular ejection fraction due to previous myocarditis. He had been under therapy with carvedilol 6.25 mg twice a day, ramipril 5 mg per day, and acetylsalicylic acid daily for 8 years. A hyperdense lesion was detected in the right transverse sinus region on cranial computed tomography (CT) imaging. Enlargement of the transverse sinus, sigmoid sinus, and jugular vein due to a hypointense thrombus mass was subsequently documented on cerebral MR imaging. Thereafter, MR venography was performed and total thrombosis of the venous sinuses and disappearance of current signal were identified (Figure 1).

**Figure 2:**
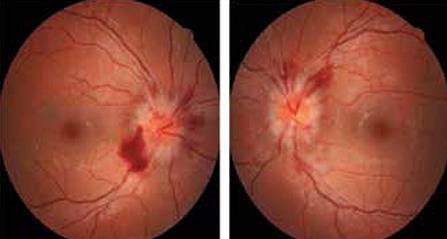
Papilledema seen in both fundus examinations

On ophthalmologic examination, the patient’s visual acuity was 20/20 without correction in both eyes. The anterior segments of both eyes were normal on slit-lamp examination. Fundoscopic examination revealed hyperemic optic disc and papilledema in both eyes (Figure 2). Abduction of the left eye was restricted (-2). There was near exotropia of 12 prism diopters (PD) and distance exotropia of 20 PD. Abducens palsy of the left eye associated with CVST was diagnosed. Single vision was restored in primary and reading position by prescribing base-out prismatic lenses of 6 PD for the right eye and 8 PD for the left eye. The patient was transferred to the Department of Neurology and medical treatment including levetiracetam 750 mg twice a day, acetazolamide 250 mg twice a day, and enoxaparin 0.6 ml subcutaneously twice a day was initiated to treat papilledema and CVST.

**Figure 3:**
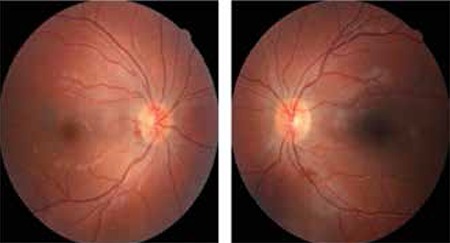
Papilledema was observed to have receded in both eyes at the 2-month follow-up examination

**Figure 4:**
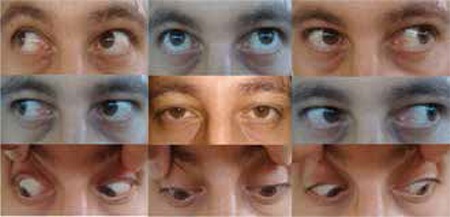
Normal gaze in all directions at the 2-month follow-up examination

Hematologic and rheumatologic laboratory tests did not reveal any underlying thrombotic disorder. In addition, the patient had no history of diabetes, hypertension, atrial fibrillation, trauma, or raised intracranial pressure. Thus, heart failure with reduced left ventricular ejection fraction was the most likely factor associated with the etiology of CVST. At the follow-up examination 2 months later, bilateral papilledema had resolved (Figure 3) and abduction of the left eye had returned to normal (Figure 4). Therefore, the use of prismatic lenses for single vision in all positions was discontinued.

## DISCUSSION

CVST presents with serious clinical signs and can be life-threatening or involve severe neurological consequences. It can be accurately diagnosed by MR venography.6 CVST commonly manifests with symptoms and signs associated with abnormally increased intracranial pressure. Headache is the most frequent symptom,7 while hemiparesthesia, hemihypoesthesia, epileptic seizures, and papilledema are frequent accompanying symptoms.^3^

Lowering of intracranial pressure is the primary goal in the treatment of idiopathic intracranial hypertension. The first-line treatment is acetazolamide, which was used for our patient.^8^ Systemic anticoagulation and chemical (tPA) and mechanical thrombolysis are the therapies for CVST.^9^ Despite the increased risk of hemorrhage, targeted endovascular delivery of thrombolytic agents may also be a treatment option.^9^ Other therapeutic measures, including steroids, antiepileptics, diuretics, hyperventilation, and shunting procedures, have also been used to manage increased intracranial pressure and seizures in CVST.^10^ Surgical thrombectomy and decompressive craniectomy should be considered, especially in young patients, when neurological deterioration occurs despite adequate anticoagulation therapy.^11^

In our case, headache, sudden-onset vomiting and nausea, epileptic seizure and papilledema were the initial signs and symptoms indicating elevated intracranial pressure. In addition, we observed diplopia and restriction of abduction associated with abducens palsy in the left eye. It is known that abducens palsy may develop due to increased intracranial pressure.^12,13^ However, the first case of abducens palsy as a consequence of CVST was reported by Marzo et al. in 2001.^14^ The case involved contralateral abducens palsy due to sigmoid sinus thrombosis complicated by chronic otitis media. This and other reported cases commonly had a local anatomic cause for the thrombotic events and their complications; our case differs mainly in the etiology, which was associated with an increased thrombosis due to heart failure with reduced left ventricular ejection fraction. The latter is accepted as a novel cardiovascular risk factor for increased thrombosis.^15^It may lead to systemic and venous thromboembolism, potentially progressing to stroke and sudden death. Impaired blood flow due to low cardiac output, endothelial dysfunction, abnormal platelet function and increased soluble vascular and endothelial pro-thrombotic factors cause a hypercoagulable milieu similar to that in Virchow’s triad, as we observed in our case.16 These mechanisms underlie the rationale for chronic anticoagulation therapy in patients with congestive heart failure. Our case provides clinical evidence of the potentially lethal consequences of thrombotic complications associated with heart failure and the necessity for chronic anticoagulation.

At the end of the 2-month follow-up period, it was observed that clinical findings related to increased intracranial pressure had completely regressed and no deviation, diplopia or restriction in abduction of the left eye remained. The papilledema of the left eye had completely resolved and the hemorrhage which had receded in the temporal of the optic disc was seen only on the right.

In conclusion, this case demonstrates that heart failure with reduced left ventricular ejection fraction may be associated with CVST, a rare but potentially lethal neurological disorder with serious clinical consequences. A high clinical index of suspicion, appropriate multidisciplinary approach, and early diagnosis and treatment can be life-saving in the management of CVST.
